# Dopamine Rebound-Excitation Theory: Putting Brakes on PTSD

**DOI:** 10.3389/fpsyt.2016.00163

**Published:** 2016-09-27

**Authors:** Jason C. Lee, Lei Philip Wang, Joe Z. Tsien

**Affiliations:** ^1^Department of Neurology, Brain and Behavior Discovery Institute, Medical College of Georgia, Augusta University, Augusta, GA, USA; ^2^Department of Psychiatry, Medical College of Georgia, Augusta University, Augusta, GA, USA

**Keywords:** stress resilience, post-traumatic stress disorder, fear memory, dopamine, fear generalization

## Abstract

It is not uncommon for humans or animals to experience traumatic events in their lifetimes. However, the majority of individuals are resilient to long-term detrimental changes turning into anxiety and depression, such as post-traumatic stress disorder (PTSD). What underlying neural mechanism accounts for individual variability in stress resilience? Hyperactivity in fear circuits, such as the amygdalar system, is well-known to be the major pathophysiological basis for PTSD, much like a “stuck accelerator.” Interestingly, increasing evidence demonstrates that dopamine (DA) – traditionally known for its role in motivation, reward prediction, and addiction – is also crucial in regulating fear learning and anxiety. Yet, how dopaminergic (DAergic) neurons control stress resilience is unclear, especially given that DAergic neurons have multiple subtypes with distinct temporal dynamics. Here, we propose the *Rebound-Excitation Theory*, which posits that DAergic neurons’ rebound-excitation at the termination of fearful experiences serves as an important “brake” by providing intrinsic safety-signals to fear-processing neural circuits in a spatially and temporally controlled manner. We discuss how DAergic neuron rebound-excitation may be regulated by genetics and experiences, and how such physiological properties may be used as a brain-activity biomarker to predict and confer individual resilience to stress and anxiety.

In describing emotions as natural selection traits, Darwin observed that fear is universal across multiple species (1). Despite its biological importance, fear can become dysregulated, such that an otherwise harmless situation or neutral cue can later trigger an unreasonable and exaggerated fearful response, resulting in psychiatric disorders, such as anxiety disorders, panic attacks, and post-traumatic stress disorders (PTSD) (2). PTSD patients exhibit avoidance behaviors, hypervigilance, and sleep disturbance. They also experience persistent negative mood and flashbacks about the traumatic event (3). Investigation into the fear circuit has revealed that PTSD could arise due to enhanced fear-learning or fear-sensitization (4, 5), reduced or delayed fear extinction (6, 7), or impaired safety-learning processes (8–10). One key criterion for PTSD diagnosis is the exposure to a traumatic or stressful event. However, it is well-known that only a small percentage of individuals develop PTSD following trauma or stressors (11). What are the neural mechanisms responsible for the inter-individual variability in stress resilience?

Indeed, the interest in stress resilience has increased in recent years. Several genetic studies have identified potential molecular contributors to stress resilience involving neural circuits, such as the serotoninergic circuit and hypothalamic–pituitary–adrenal axis (12–17). Interestingly, resilience and susceptibility to a stressor, such as using social defeat protocols in rodents, have also been attributed to the mesolimbic dopamine (DA) circuit (18). However, how dopaminergic (DAergic) neural activities on a network level contribute to resilience or susceptibility remains unclear. Here, we wish to propose the *Rebound-Excitation Theory*, which posits that the DAergic neuromodulatory circuit generates spatially and temporally precise safety signals upon the termination of fearful stimuli, which act as important innate brakes on fear signals in the brain. Importantly, this intrinsic rebound-excitation signal can be modified by repeated exposure to aversive experiences, as well as by associative safety-signal learning via pairing with conditioned stimulus (CS, such as a neutral tone), via NMDA receptors on DAergic neurons.

## DA Circuit Diversity and Complexity

DAergic neurons are well-known to subserve a wide range of biological functions, such as learning and memory (19), motivation (20, 21), reward prediction error (22, 23), salience and valence (24, 25), addiction (26, 27), and wanting (28, 29). Recently, a growing body of evidence suggests that DA may also play a crucial role in regulating fear memory and behaviors (30–34). Micro-dialysis and fast-scan cyclic voltammetry studies have also shown that DA concentrations change in DAergic projection areas, such as the nucleus accumbens (NAc), in response to aversive stimuli (32, 35). Moreover, *in vivo* electrophysiological studies in rodents and monkeys have also reported heterogeneous DAergic responses to aversive events (24, 36–43). However, understanding how DAergic neurons subserve fear processing is proving to be a difficult task. For instance, DAergic neurons exhibit both tonic and burst type firing modes (44–46), both of which exert distinct DA release profiles that act on separate DA receptor populations (47). Moreover, DAergic neurons are diverse in nature and can be classified by multiple criteria, such as anatomical locations (48), input-projections (49–52), distinct response dynamics to rewards, and aversive stimuli (25, 43, 53). Adding more dimensions to the circuit complexity, DAergic firing exhibits temporal and spatial dynamics that must also be taken into consideration. For example, multi-phasic temporal dynamics in DAergic neurons have been reported in studies using unexpected (unconditioned) aversive stimuli (40, 53). Consistent with such complex dynamics, we recently described computational classifications of DAergic subtypes based on their distinct inter-spike-interval dynamics (54). Such classifications were further verified by optogenetic methods (54). In addition, downstream targets receiving DAergic projections can send feedback projections to modulate DA activities (55–57). Likewise, local controls of DAergic activities by GABAergic neurons can further add to the complexity of DA signal regulation (58–60).

## Rebound-Excitation Theory

In order to understand the role that DAergic neurons play in processing traumatic experiences, the aforementioned DA circuit complexity necessitates the need to systematically compare and contrast how distinct subpopulations of DAergic neurons respond to emotionally traumatizing events. Emerging evidence clearly suggests that DAergic neurons readily respond to aversive stimuli, including air-puffs to the eyelids of monkeys, or administering bitter tastant quinine in awake rats or tail pinches or foot-shocks to anesthetized rats. However, how the same DAergic neurons respond to a variety of fearful stimuli has rarely been investigated. Thus, the tuning properties of distinct DAergic neuron subtypes remain unclear. Moreover, anesthetized states examined in some of the literature could alter the hedonic state of the stimuli and thus the neural responsivities (61, 62). Furthermore, while negative stimuli – such as air-puffs to the eye or administration of quinine to the mouth, or tail pinch under anesthetized state – are aversive in nature, they are not appropriate as PTSD-inducing models.

**Figure 1:**
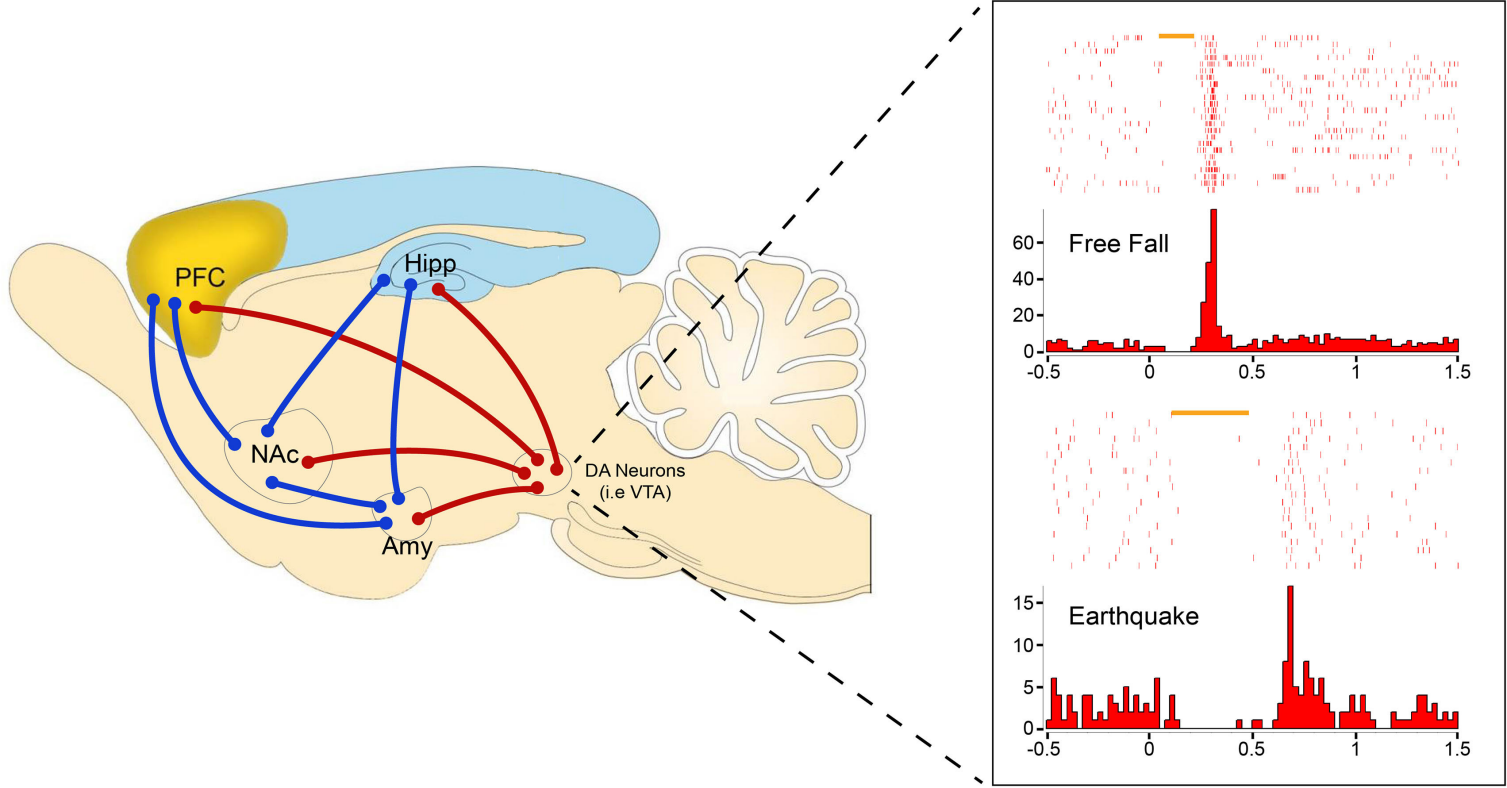
**Representative amygdalar fear circuits (blue) with DA circuit (red) involvement**. DA units showing rebound-excitation to two distinct types of fearful stimuli: free fall (top) and earthquake (bottom). Rebound-excitation occurs at the termination of fearful stimuli and is proposed to serve as an innate safety signal to modulate fear-related learning and behaviors by broadcasting to downstream targets such as the amygdala (Amy), nucleus accumbens (NAc), prefrontal cortex (PFC), or hippocampus (Hipp).

To specifically examine how DAergic neurons respond to traumatic fear in real-life events, we used laboratory versions of fearful unconditioned stimuli (US) (such as an earthquake, free fall, or foot-shocks) that induce profound fear memory and rapid cardiac responses in freely behaving mice (63). Combined with pharmacological and optogenetic methods, chronic *in vivo* recordings of VTA DAergic neural activities in freely behaving mice have shown two major types of DAergic neuron responses: fear-inhibited and fear-excited DAergic neurons (40). Notably, we observed that many aversive-inhibited DAergic neurons show phasic rebound-excitation responses at the offset of unexpected aversive stimuli (40) (Figure 1). This unique response pattern to fearful US has lent us the idea that offset phasic rebound-excitation of this particular sub-population of DAergic neurons may act as a critical safety signal to encode the termination of a fearful event. The signal strength of this phasic DA release, time-locked to the termination of fearful events, will exert immediate as well as long-term changes in downstream targets, thereby setting up the different thresholds for each individual’s resilience to stress and anxiety.

## Testing the Rebound-Excitation Theory Experimentally

The *Rebound-Excitation Theory* predicts that attenuation or lack of rebound safety signals following fearful stimuli leads to stress susceptibility, whereas strong rebound safety signals confer stress resilience. Furthermore, the *Rebound-Excitation Theory* predicts that a rebound safety signal is likely to be evolutionarily conserved across multiple species and is subject to modulation via experience-dependent synaptic plasticity. One of the most powerful ways to study stress resilience is to directly examine individual stress response variability following a stressor (64). As a result, testing for rebound-excitation in human PTSD vs. trauma resilient populations could prove invaluable. However, present imaging techniques, such as functional magnetic resonance imaging (fMRI) and EEG, have limited temporal and spatial resolution, which makes rebound-excitation study in humans difficult. This may change with development of transformative BRAIN technologies in future.

On the other hand, large-scale *in vivo* electrophysiological recordings in freely behaving laboratory animals now allows single neural unit activity to be accessed in real time with high temporal and spatial resolution (65). In addition, reliable identification of DAergic neuron subtypes can be achieved by using optogenetics and computational analysis (54). Therefore, initial efforts to test the *Rebound-Excitation Theory* may be fruitful in animal models. Much like that of the human population, a fraction of wild-type laboratory animals are known to be more susceptible to stressors than others (17, 66, 67). Thus, the *Rebound-Excitation Theory* can be initially tested by screening and comparing DA rebound-excitation in stress-susceptible vs. stress-resilient animals.

Genetic manipulation in laboratory animals that alters rebound-excitation safety signals can also be useful. For example, we have produced DA neuron-specific NMDA receptor knockout (DA-NR1-KO) mice (68) and have shown that the NMDA receptors in DAergic neurons play a critical and specific role in regulating phasic firing patterns of the DAergic neurons (69). Given the reported excessive fear generalization in this mutant model (41), this knockout model offers a rare opportunity to examine the circuitry dynamics by which DAergic neuron NMDA receptors modulate rebound-excitation safety signals and fear behaviors. This mouse model can also be used to test whether repeated exposure to aversive US may enhance rebound-excitation-based safety-signals in normal animals vs. little or no enhancement effect on PTSD-sensitive models.

## Targets and Actions of DAergic Neuron Rebound-Excitation

What are the potential mechanisms through which DA rebound-excitation safety signals alleviate fear and fear overgeneralization? In general, this innate safety signal can come from three major sources of regulation: (1) the intrinsic DAergic neuron properties, such as ion channels and receptor compositions that produce rebound phasic firings; (2) downstream targets that detect and process DAergic neuron rebound excitation; and (3) cortical and subcortical feedback to the DA circuits.

Obviously, DAergic neurons’ safety signals may directly modulate downstream targets’ neural and biochemical activities. Phasic firing by DAergic neurons can result in robust DA release (70, 71), leading to the elevation of DA in a variety of neural circuits [i.e., the prefrontal cortex (PFC), striatum, amygdala, hippocampus, etc.]. For example, DA is known to induce short-lived excitatory responses via D1 receptors in downstream neurons (72). The time window in which DA mediates structural changes, such as dendritic spine enlargement is also precise, in the range of seconds or less (0.3–2 s) (73). Furthermore, manipulating DAergic firing has been shown to produce acute behavioral changes (74, 75). Moreover, DA is known to be involved in the induction and maintenance of long-term potentiation (LTP) in the amygdala and hippocampus, respectively (76, 77). DAergic neurons are well-known to project to the PFC, which is important for processing emotional information (78). For example, we recently showed that neurons in the anterior cingulate cortex exhibited diverse responses in response to traumatizing events, such as mild blast events, which mimicked the combat experiences of war fighters when witnessing an explosion of a road-side bomb (79). Importantly, we showed that robust-pattern reverberation occurs frequently in the ACC of blast-exposed animals (79). Pattern reverberation is a process by which real-time memory patterns and traces are replayed shortly after emotionally charged, episodic events (80, 81). DA rebound-excitation may modulate pattern reverberation of fearful memories in downstream targets, such as the ACC and hippocampus. Abnormal pattern reverberation due to alteration in DA rebound-excitation may manifest as PTSD symptoms, such as flashbacks. Effects of DA rebound-excitation on real-time memory traces can be examined using large-scale recording and decoding methods (80, 82). In addition, a DA signal may modulate adult neurogenesis in the dentate gyrus (83–86), which has been linked to stress and depressive behaviors (87, 88), as well as to reduced clearance of fear memory traces (89).

Furthermore, changes in rebound-excitation-based intrinsic safety signals can likely manifest at multiple circuit levels given the DA circuit complexity. For instance, dysregulation from upstream DAergic afferent inputs (90) may alter rebound-excitation safety signals, perhaps by influencing local GABAergic control within the VTA. Dysfunction in feedback control from cortical and subcortical sites (55–57) may also cause pathological alterations in rebound-excitation and fear-related behaviors. This can be highly interesting because cortical and subcortical inputs to DAergic neurons can serve as an important mechanism to create Pavlovian learning paradigms under which associative safety-learning can occur. This would enable a set of Pavlovian neural substrates – which have been extensively studied under Prediction Error theory and temporal difference (TD) models – to be recruited to generate CS/US pairing-triggered safety-learning signal (which is distinct from the innate rebound-excitation-mediated safety signals as we described here). Moreover, abnormal expression of DA receptors or mutations in DA receptors’ intracellular signal transduction may lead to instances in which the rebound-excitation of DAergic neurons is intact but is unable to activate downstream targets.

Another possible physiological effect of DA is to regulate neural network synchronization and oscillation. Neural synchronization and oscillation are thought to be an important mechanism by which networks of neurons coordinate their activities in a temporally meaningful pattern to generate cognition, perception, and behaviors (91–93). One study examining cortical input to the hippocampus and Schaffer-collateral found that DA can modulate the excitatory drive onto pyramidal and GABAergic interneurons (94). Additionally, therapeutic dosage of DA agonist levodopa has been shown to cause the frequency synchronization between the globus pallidus and subthalamic nucleus to shift from low frequency (<30 Hz) to high frequency (~70 Hz) (95). Moreover, recent studies using neuroimaging techniques, such as magnetoencephalography (MEG) and functional magnetic resonance imaging (fMRI), have found irregular network synchrony and oscillations in PTSD patients (96, 97). Therefore, rebound-excitation of DAergic neurons on modulating fear circuits should be investigated at multiple levels.

## Rebound-Excitation Theory Offers a New Approach to Study PTSD

The *Rebound-Excitation Theory* predicts that rebound-excitation consistency across multiple fearful experiences may, in part, account for inter-individual variability in stress resilience. We have previously observed rebound-excitation to be similar between distinct fearful events (40). Therefore, a stress resilience index may be constructed by accessing rebound-excitation in individual subjects, and such a resilience index may serve as a useful predictor in clinical settings to screen individuals that may be stress-susceptible. Indeed, we have recently developed fear resistance indices in mice based on inter-individual variability in cardiac responses [heart rate variability (HRV)] across multiple fearful experiences (63). Given that PTSD patients had abnormal HRV (98), in the future, it will be of great interest to study the correlation between inter-individual variability of rebound-excitation signals and HRV. Such potential correlation may provide a mechanistic framework to examine predictive values of HRV in the human population.

Moreover, the proposed theory should open new avenues to develop novel therapeutic strategies for studying and treating PTSD. For instance, DA burst firing has been shown to increase at the onset and offset of voluntary exercises (39). Therefore, exercise with an appropriate time regimen might be explored as a way to improve behavioral therapy. In fact, exercises can enhance neurogenesis in the hippocampus (99), a process linked with reducing depression (88, 100, 101). It is encouraging that a pilot study in an adolescent with PTSD showed that aerobic exercises reduced the symptoms of PTSD (102). Rebound-excitation signals can also be used as a brain-activity biomarker to screen novel compounds for their *in vivo* drug efficacies in preclinical PTSD research.

In literature, external CS (such as a neutral tone) have been used to create Pavlovian association and turning CS into the predictive safety-learning cues about signaling the absence of fearful US in animal models (8, 10, 103–106). This powerful associative learning process utilized Pavlovian conditioning paradigms by repeated pairing of CS with US. Interestingly, PSTD models and patients exhibit impaired ability to suppress fear response even in the presence of conditioned safety-learning cues, despite they can learn normally in Pavlovian fear conditioning (9). This suggests that PTSD deficit was not a result of simple failure in associative learning, but rather specific defects in generating innate safety signals as well as prediction errors based on extinction or discrimination learning. It further highlights the need to differentiate the neural mechanisms underlying conditioned safety-learning of external neutral cues vs. the safety signals derived from DA rebound excitation. It would be of great interest to examine how DAergic neuron rebound excitation signal interacts and influences external safety-learning process, or vice versa, and whether such associative dynamics can be further modeled by prediction error based on TD learning model (23, 107). Because real-life traumatic events rarely occurred by the predictive CS, DA rebound-excitation theory now offers a novel approach to analyzing innate DA safety signal in response to unpredictable US, thereby leading explanation as to how the brain can taper down the otherwise excessive neural trace reverberation that typically followed upon fear experiences (79–82).

This critical distinction between the proposed rebound-excitation theory and TD model should and can be tested experimentally; for example, DAergic neuron rebound-excitation should be observed upon US stimulation alone without repeated CS/US pairing. Rebound-excitation signals the termination of the aversive US itself. As a result, variations in stimulus durations can be used, together with repeated trials, to further define dynamic modulation of its rebound responses, in a similar way that TD learning model and prediction error theory have been examined. Because repetition of aversive US can lead to varying degrees of behavioral habituation or sensitization, we postulate that the repeated presentation of US over trials will lead to stronger DAergic neuron rebound-excitation signal in PTSD-resilient animals vs. diminished rebound-excitation in PTSD-prone animals, and this process should be dependent on the NMDA receptors of the DAergic neurons. It is conceivable that this intrinsic safety signal based on DAergic neuron rebound-excitation is advantageous for an organism’s overall survival given the unpredictability of aversive stimuli in nature in terms of types, duration, as well as intensity. Defects in this innate safety-signal due to genetic mutations in the relevant circuits can make the animals vulnerable to PTSD and impair safety-learning in general. Better understanding of both the innate safety-signaling mechanisms, gene mutations, and Pavlovian condition-based safety-learning mechanisms can lead to novel insights to PTSD pathogenesis.

In summary, the proposed *Rebound-Excitation Theory* specifies that DAergic neurons generate intrinsic safety signals at the termination of unconditioned fearful events in a spatially and temporally precise manner. Impairment in the production and reception of this safety signal constitutes a potentially genetic defect in the brake on the fear system. Restoration of this rebound-excitation signal may offer a much-needed new avenue for developing pharmacological and behavioral therapeutic strategies to treat psychiatric disorders.

## Author Contributions

JL and JT developed the idea and worked with LW. JL generated the figure with input from LW and JT. JL, LW, and JT co-wrote the article.

## Conflict of Interest Statement

The authors declare that the research was conducted in the absence of any commercial or financial relationships that could be construed as a potential conflict of interest.
